# Membrane TLR9 Positive Neutrophil Mediated MPLA Protects Against Fatal Bacterial Sepsis

**DOI:** 10.7150/thno.37139

**Published:** 2019-08-14

**Authors:** Zhaogang Yang, Luowei Wang, Hongmei Yu, Ruonan Wang, Yawei Gou, MingMing Zhang, Chen Kang, Tongzheng Liu, Yu Lan, Xiaobing Wang, Jiwei Liu, Merideth A. Cooper, Xin Li, Kai Yue, Yongli Yu, Liying Wang, Betty Y.S. Kim, Wen Jiang, Wei Sun

**Affiliations:** 1 Department of Molecular Biology, College of Basic Medical Sciences Jilin University, Changchun 130021, China; 2 Department of Radiation Oncology, The University of Texas Southwestern Medical Center, Dallas, TX 75390, USA; 3 China-Japan Union Hospital, Jilin University, Changchun, Jilin 130021, China; 4 Carver College of Medicine, University of Iowa, Iowa City, IA 52242, USA; 5 Jinan University Institute of Tumor Pharmacology, Guangzhou 510632, China; 6 Tumor Marker Research Center, Cancer Institute and Hospital, Chinese Academy of Medical Sciences and Peking Union Medical College, Beijing 100021, China; 7 Department of Immunology, College of Basic Medical Sciences, Jilin University, Changchun 130021, China; 8 Tumor Three Wards, Nanyang Central Hospital of Henan Province, Nanyang, Henan, 473000, China; 9 Department of Neurosurgery, The University of Texas MD Anderson Cancer Center, Houston, TX 77030, USA

**Keywords:** sepsis, neutrophils, TLR9, caveolin-1, preconditioning, MPLA

## Abstract

Sepsis is a major cause of patient mortality and morbidity from bacterial infections. Although neutrophils are known to be important in the development of sepsis, how distinctive neutrophil subtypes regulate inflammatory processes involved in septicemia remains unclear. Preconditioning protects organisms against subsequent higher-dose exposures to the same, or even different, stimuli. Several studies have reported various effects of preconditioning on immune cells. However, the detailed mechanisms underlying neutrophil-mediated protection through preconditioning in sepsis remain unknown.

**Methods**: Flow cytometry was conducted to sort the mice peritoneal lavage cells and the blood samples from patients with sepsis. Western blotting and ELISA were carried out to elucidate the expression of TLR9 signal transduction pathway proteins. Histological analysis was used to assess the effect of InP on intestine and liver structure in *tlr9^-/-^* and *cav-1^-/-^* mice. Fluorescence microscopy, Co-IP, and FRET were carried out to determine the association of TLR9 with Cav-1.

**Results**: We show that membrane toll-like receptor-9 positive (mTLR9^+^) neutrophils exert a protective effect against fatal bacterial infections through the process of inflammatory preconditioning (InP). InP, which occurs in the setting of a low-dose bacterial challenge, active ingredient is Monophosphoryl lipid A (MPLA), triggers the membrane translocation of TLR9 from the neutrophil cytosol, where it binds to Cav-1. Our findings showed that InP enables TLR9 to facilitate MyD88-mediated TRAF3 and IRF3 signal transduction. Depletion of either TLR9 or Cav-1 largely eliminates the neutrophil-mediated InP effect in sepsis models *in vitro* and *in vivo*. Further, examination of clinical samples from patients with sepsis showed that clinical outcomes and likelihood of recovery are closely correlated with mTLR9 and Cav-1 expression in circulating neutrophils.

**Conclusion**: These results demonstrate that the TLR9-Cav-1 axis is a critical signaling pathway involved in the regulation of neutrophil-dependent MPLA mediated InP, and the presence of mTLR9^+^ neutrophils could be an attractive indicator of clinical outcomes in bacterial sepsis that could be further explored as a potential therapeutic target.

## Introduction

Bacterial sepsis is a major cause of morbidity and mortality from infection worldwide. During sepsis, neutrophils, the first wave of infiltrating immune cells, migrate to sites of infection, where they are activated and destroy invading microorganisms. Impaired migration of neutrophils to infection sites is associated with poor therapeutic outcomes in sepsis 1, 2, indicating that neutrophils are beneficial in protecting a host from developing severe sepsis. However, the detailed mechanisms underlying neutrophil-mediated protection in sepsis remain unknown.

Recently, membrane Toll-like receptor 9 (mTLR9)-expressing neutrophils have been identified in human and mice 3, 4. These mTLR9^+^ neutrophils can sense bacterial DNA released during sepsis, as well as mitochondrial DNAs released during severe trauma, to activate the p38 MAP kinase (MAPK) pathway 5, resulting in acute lung injury as a part of the systemic inflammatory response syndrome^4^, ^5^. Several studies reported that mTLR9^+^ neutrophils are involved in excessive inflammatory responses and exacerbated tissue injuries in the setting of fulminant sepsis 6-8. In this study, we discovered that the exact role of mTLR9^+^ in the setting of bacterial infection is much more dynamic than previously described, and these cells may in fact have a protective effect against subsequent lethal sepsis if an organism had been exposed to a prior low-dose bacterial pathogen (“preconditioning”).

Preconditioning protects organisms against subsequent higher-dose exposures to the same, or even different, stimuli. Several studies have reported various effects of preconditioning on immune cells. For instance, endotoxin preconditioning leads to renal epithelial protection in different *in vivo* models of sepsis via macrophages and is critically involved in renal protection 9-12. Hypoxia also preconditions the innate immune response and determines survival outcomes after bacterial infection through suppression of HIF-1α and neutrophil metabolism 13. Preconditioning of mesenchymal stem cells *ex vivo* by hypoxia, inflammatory stimuli, or other insults can prepare them to endure extreme conditions and improve their regulatory functions on local immune responses 14, 15.

Lipid A is from a rough strain *E. coli* lipopolysaccharide using treatment with mild acid and heat followed by chromatography. Lipid A contains up to 7 fatty acid side chains. The average molecular weight is 1.7-1.8 kDa, depending on the number and identity of fatty acid chains present. Lipopolysaccharides are composed of a hydrophobic lipid (lipid A), a hydrophilic core polysaccharide chain, and a hydrophilic O-antigenic polysaccharide side chain. Lipid A did not induce necrosis or regression of tumors in mice. Lipid A is of great pathophysiological interest since it exerts many profound effects when injected into animals, including the induction of endotoxic shock, pyrogenicity, macrophage activation, B lymphocyte mitogenicity, induction of interferon production complement activation, and tumor regression. Monophosphoryl lipid A (MPLA) is nontoxic, whereas diphosphoryl lipid A is toxic. MPLA has been used in the preparation of liposomes for antigenic studies and is a component of the Ribi Adjuvant System 16.

In the present study, we found that mTLR9^+^ neutrophils could protect mice from sepsis induced by intraperitoneal (*i.p.*) injection of *E. coli* when the animals had previously been infected with MPLA. This inflammatory preconditioning (InP) effect was associated with the recruitment of mTLR9^+^ neutrophils to the peritoneal cavity, and mice treated with lavage fluid containing mTLR9^+^ neutrophils survived exposure to *E. coli* at a lethal dose. These results suggested that MPLA could precondition mice by recruiting mTLR9^+^ neutrophils, subsequently preparing the mice for acquiring resistance to lethal infections. We next sought to identify the molecular mechanisms by which mTLR9^+^ neutrophils protect the organism against sepsis. Finally, we demonstrate that clinical outcomes in patients with bacterial sepsis are strongly correlated with the levels of mTLR9^+^ neutrophils in systemic circulation, thus providing rationale for its use in diagnosing and treating sepsis. These results reveal important insights into the complex role neutrophils play in regulating septic responses.

## Results

### Neutrophil-mediated MPLA inflammatory preconditioning (InP) protects mice from lethal dose of bacteria

**Figure 1:**
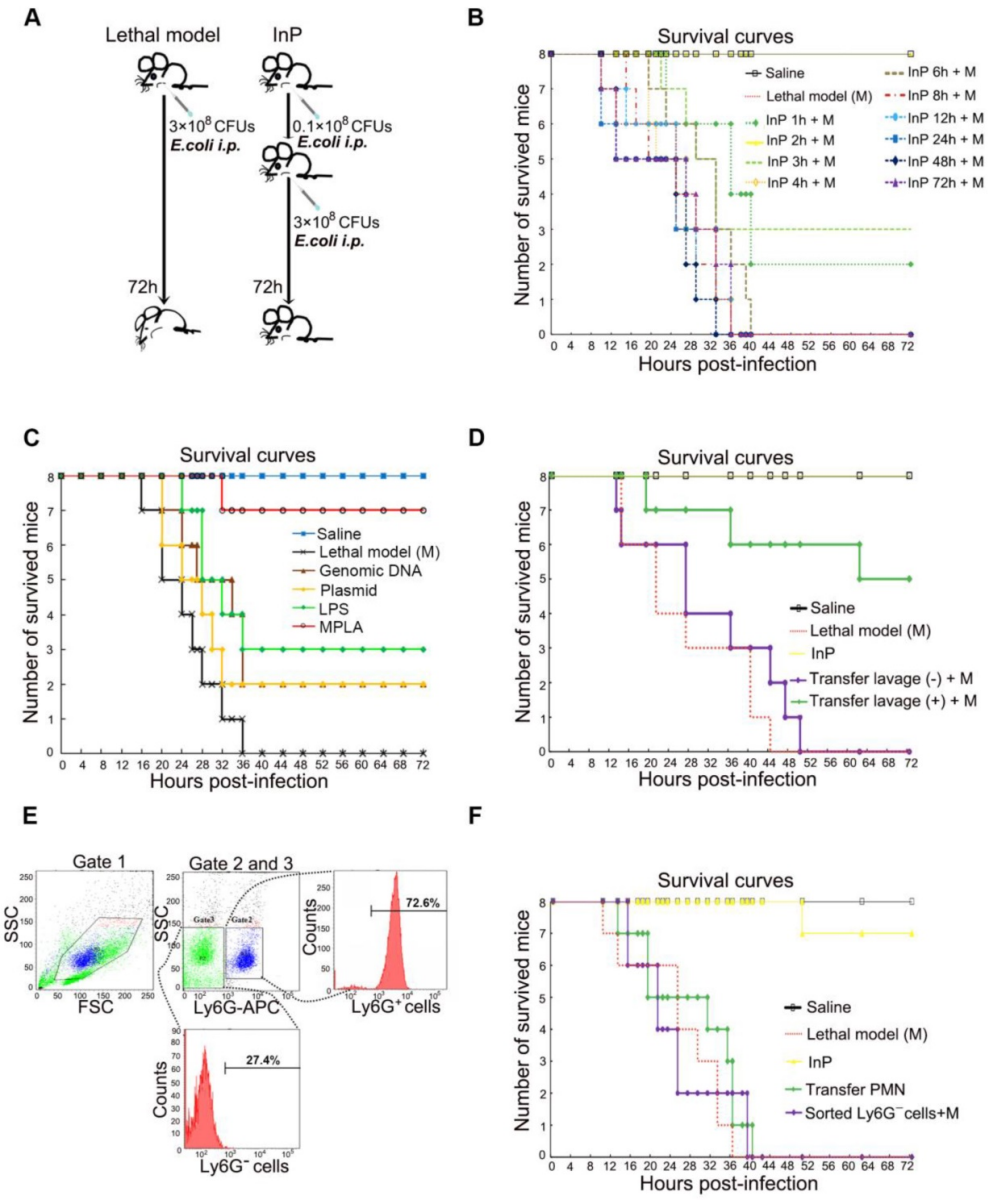
** The effect of MPLA mediated inflammation preconditioning (InP) on mice. (A)** Experimental procedures. **(B)** Survival curves. Each line represents survival of mice in groups of 8 mice per group. The mice received 0.1×10^8^ CFU *E. coli* firstly, then were injected with the lethal dose of *E. coli* (3×10^8^ CFUs) respectively at different time points, * *vs.* Saline.** (C)** Survival curves. Each line represents survival of mice in groups of 8 mice per group. The mice received different parts of *E. coli* firstly, then were injected with the lethal dose of *E. coli* (3×10^8^ CFUs) respectively at 2h, * *vs.* Saline.** (D)** The survival curves. The mice received 0.1×10^8^ CFU *E. coli* firstly, and the collected peritoneal lavage was transferred into another mouse to see the protection of peritoneal lavage. And both mice were then *i.p.* injected the lethal dose of *E. coli* (3×10^8^ CFUs). **(E)** Cell sorting of peritoneal lavage cells. **(F)** The survival curves. The mice received 0.1×10^8^ CFUs *E. coli* firstly, then the sorting cells were transfer into another mouse, and both mice were then *i.p.* injected with the lethal dose of *E. coli* (3×10^8^ CFUs).

Sepsis is caused by an immune response triggered by an infection, which in most cases is bacterial. Preconditioning is an important intrinsic process that protects the organism against subsequent stronger exposures to the same or different stimuli. However, it is unclear whether the protective effect of preconditioning also applies to sepsis, and if so, which processes are involved. To answer the first question, we established a lethal inflammation model by intraperitoneal (*i.p.*) injection of 1 mL *E. coli* (3×10^8^ CFU/mL) in mice (Figure 1A-B). As expected, all *E. coli* injected mice died within 72 h, consistent with previous reports 8. We next evaluated the effect of prechallenging mice with *E. coli*, otherwise known as inflammatory preconditioning (InP), at different time points before a lethal dose of *E. coli* was given. Unexpectedly, we found that the survival was greatly increased when mice received a low dose of *E. coli* (0.1×10^8^ CFU) before the lethal dose (Figure 1A-B). Postmortem tissue analyses showed severe intestine and liver damage in the lethal dose treatment-only group, whereas mice with InP had significantly less intestine and liver damage (Figure S1A). Since InP at 2 h before the lethal dose resulted in the highest survival rate, this condition was chosen for subsequent experiments.

In order to determine the active ingredient of InP induced by *E. coli* (0.1×10^8^ CFU), we used *E. coli* genomic DNA, plasmid, lipopolysaccharide (LPS) and attenuated chemically modified lipopolysaccharide monophosphoryl lipid A (MPLA) to carry out InP induction experiments. The survival rates showed that genomic DNA and plasmid had no protective effect while LPS can only slightly increase the survival rate. In contrast, we found that the survival was greatly increased when mice received MPLA before the lethal dose (Figure 1C).

We conducted a systematic large-scale analysis of peritoneal lavage cells after *i.p.* injection of MPLA, and found increases in the numbers of B cells, T cells, and neutrophils in InP mice relative to the control mice; neutrophils in particular showed a 4-fold increase (Figure S1D). Therefore, we hypothesized that neutrophils within the peritoneal lavage fluid are likely to be a major factor contributing to the protective effect of InP. To test this hypothesis, we collected peritoneal lavage samples from group (A) mice, which had received MPLA via *i.p.* injection for 2 h, and immediately injected the collected samples into group (B) mice. Both groups of mice were then given a lethal dose of *E. coli* (3×10^8^ CFU). The mice from group (A) died within 72 h, but the group (B) mice (which had received the peritoneal lavage from group (A) mice) tolerated the lethal dose and most of them survived (Figure 1D and S1B). Again, tissue analyses revealed severe intestine and liver damage in group (A) mice, with little such damage in group (B) mice (Figure S1C).

Since Ly6G is a well-known cell-membrane protein marker of neutrophils 17, to further validate the potential role of neutrophils in InP, neutrophils were sorted by selection for Ly6G^+^ cells and the non-neutrophils were sorted by selection for Ly6G^-^ cells in the lavage fluid (Figure 1E). Because of the nature of neutrophils 17, 18, the Ly6G^+^ cells were not able to survive for long periods *ex vivo* after sorting by flow cytometry 1, 2, 4. Therefore we sorted out Ly6G^-^ cells from the lavage samples of group (A) mice as stated above, and injected the Ly6G^-^ cells into the second group of mice, followed by a lethal dose of *E. coli*. Consistent with our previous peritoneal lavage injection experiment, all mice injected with Ly6G^-^ cells died after the lethal dose of *E. coli* (Figure 1F). Collectively, these results suggest that InP/MPLA could protect the mice from subsequent lethal bacterial infection. Moreover, our findings suggested that neutrophils in the lavage fluid were likely to be responsible for the protective effect mediated by InP.

### TLR9 activation is responsible for the InP effect

**Figure 2:**
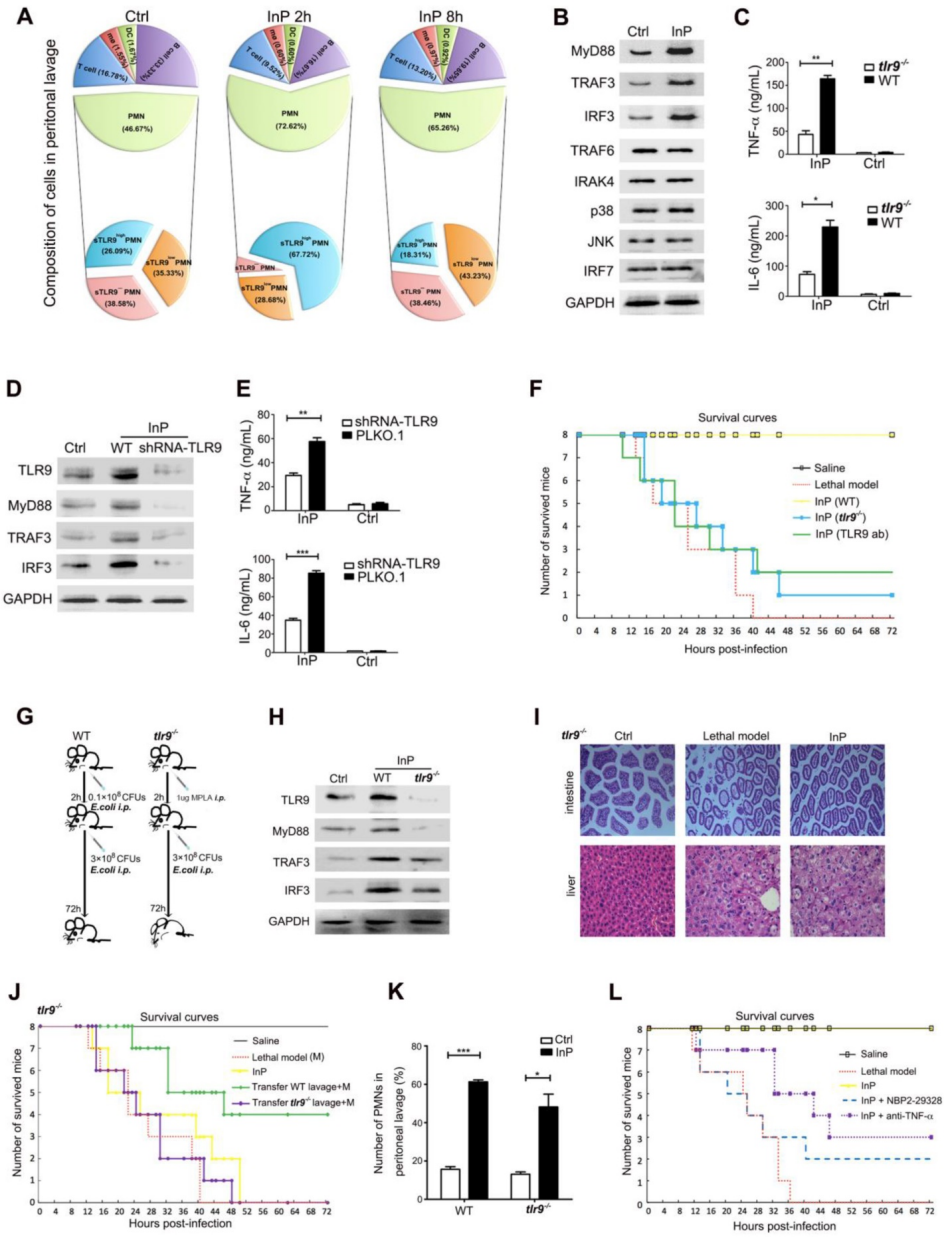
** The effect of TLR9 in MPLA mediated inflammation preconditioning (InP). (A)** The composition of peritoneal lavage cells of the mice in InP group by flow cytometry.** (B)** Western blots of total cellular from 4 independent experiments.** (C)** Enzyme-linked immunosorbent assay (ELISA) of TNF-α/IL-6 in the supernatants of peripheral blood neutrophils in mice, stimulated with 0.1×10^8^ CFUs *E. coli* for 2 h. **P<0.01 (Student's *t* test).** (D)** Expression of proteins in HL60 cells stably transfected with TLR9 shRNA and PLKO.1 (control vector), stimulated with 0.1×10^8^ CFUs *E. coli* for 2 h.** (E)** ELISA of TNF-α/IL-6 in the supernatants of HL60 cells stably transfected with TLR9 shRNA and control vector, stimulated with 0.1×10^8^ CFUs* E. coli* for 2 h. **P<0.01. **(F)** Procedure of the experiment. The WT mice were injected *i.p.* with 0.1×10^8^ *E. coli,* followed 2 h later by 3×10^8^ *E. coli,* and all 8 mice were survived (InP). TLR9^-/-^ mice were injected *i.p.* with 0.1×10^8^ *E. coli,* followed 2 h later with 3×10^8^ *E. coli,* and all 8 mice died. **(G)** Survival curves. Each line represents survival of mice in groups of 8 *tlr9^-/^*^-^ mice. **(H)** Expression of proteins in total cellular lysates of peritoneal lavage cells in WT and *tlr9^-/^*^-^ mice. **(I)** Intestine and liver tissues of *tlr9^-/^*^-^ mice with H&E staining. **(J)** The numbers of peripheral mononuclear cells in peritoneal lavage samples from WT *vs. tlr9^-/^*^-^ mice. **(K)** Survival curves, n= 8 mice per group.

To evaluate how the lavage fluid induced the InP effect, we next examined the composition of the lavage cells from InP mice. We found that most of the lavage cells were neutrophils (72.6%), and the percentages of other cells were very low (0.6% for macrophages, 0.6% for dendritic cells, 16.7% for B cells and 9.5% for T cells) 2h after InP (Figure 2A). Among neutrophils, 67.7% had high membrane TLR9 (mTLR9^high^) expression, 28.7% had low (mTLR9^low^) expression and 3.6% lacked mTLR9 expression. Interestingly, at InP 8h time point, no significant difference in cell composition was observed compared to 2h. However, the neutrophils with mTLR9^high^ expression decreased dramatically (Figure 2A). Meanwhile, MyD88, TRAF3, and IRF3, downstream effectors of TLR9, were significantly increased in InP mice compared with the control mice (Figure 2B), as were levels of the inflammatory cytokines tumor necrosis factor-alpha (TNF-α) and interleukin-6 (IL-6) (Figure 2C).

Because most neutrophils in InP group were mTLR9^high^, and because TLR9 has been shown to mediate cellular response to bacteria to mount an innate immune response 5, 9, 14, we hypothesized that activation of the TLR9 signal pathway is critical for InP. To test the hypothesis, we performed knocked down TLR9 in HL60 cells by using shRNA and investigated the effects on InP. TLR9 knockdown (KD) significantly inhibited InP-induced MyD88 and TRAF3 expression (Figure 2D) and decreased the production of TNF-α and IL-6 *in vitro* (Figure 2E). When we treated HL-60 neutrophilic promyelocytes with CpG ODN, a well-known TLR9 receptor agonist, we found drastic increases in TNF-α and IL-6 levels, an effect that was abrogated in the TLR9 KD cells (Figure S2A). We then confirmed the role of TLR9 in InP-mediated protection *in vivo* as follows. First, we found that InP had no effect on the survival of TLR9 knockout (*tlr*9^-/-^) mice, with only 1 mouse alive at 72 h after the *E. coli* attack (Figure 2F and 2G). Blocking of TLR9 on neutrophil surface by administration of TLR9 antibody after preconditioning in wild-type (WT) mice can also significantly abolish the InP effect *in vivo* (Figure 2F). Moreover, expression of MyD88 and TRAF3 was markedly decreased in *tlr*9^-/-^ mice compared with WT mice (Figure 2H), with significant liver and intestine damage noted in the *tlr*9^-/-^ animals (Figure 2I). In contrast, liver and intestine damage was largely absent in WT mice (Figure S2B). Levels of TNF-α and IL-6 were also reduced in the *tlr*9^-/-^ mice relative to the WT mice, although they were still elevated compared with the control (Figure 2C). Reintroduction of neutrophils from WT mice lavage fluid after preconditioning into *tlr9*^-/-^ mice could partially restore its InP effect in *tlr9*^-/-^ mice (Figure J). Interestingly, no difference was noted in the number of neutrophils between *tlr*9^-/-^ and WT lavage samples (Figure 2K), indicating that activation of the TLR9 signal pathway within the neutrophils is essential for the protective role of InP in sepsis, rather than the actual number of neutrophils at the infection site.

To further investigate whether the downstream TLR9 signal pathway is important for the protective role of InP, we injected either the MyD88 inhibitor NBP2-29328 or an anti-TNF-α antibody at the beginning of InP. TLR9 blockade with either one of these agents resulted in significant mouse death after a lethal challenge (Figure 2L). These results, together with data above (Figure 2H), suggest that TLR9 promote InP mediated sepsis protection via the activation of MyD88 and TNF-α.

### InP triggers the translocation of TLR9 from cytoplasm to membrane and promotes the interaction between TLR9 and Caveolin-1

**Figure 3:**
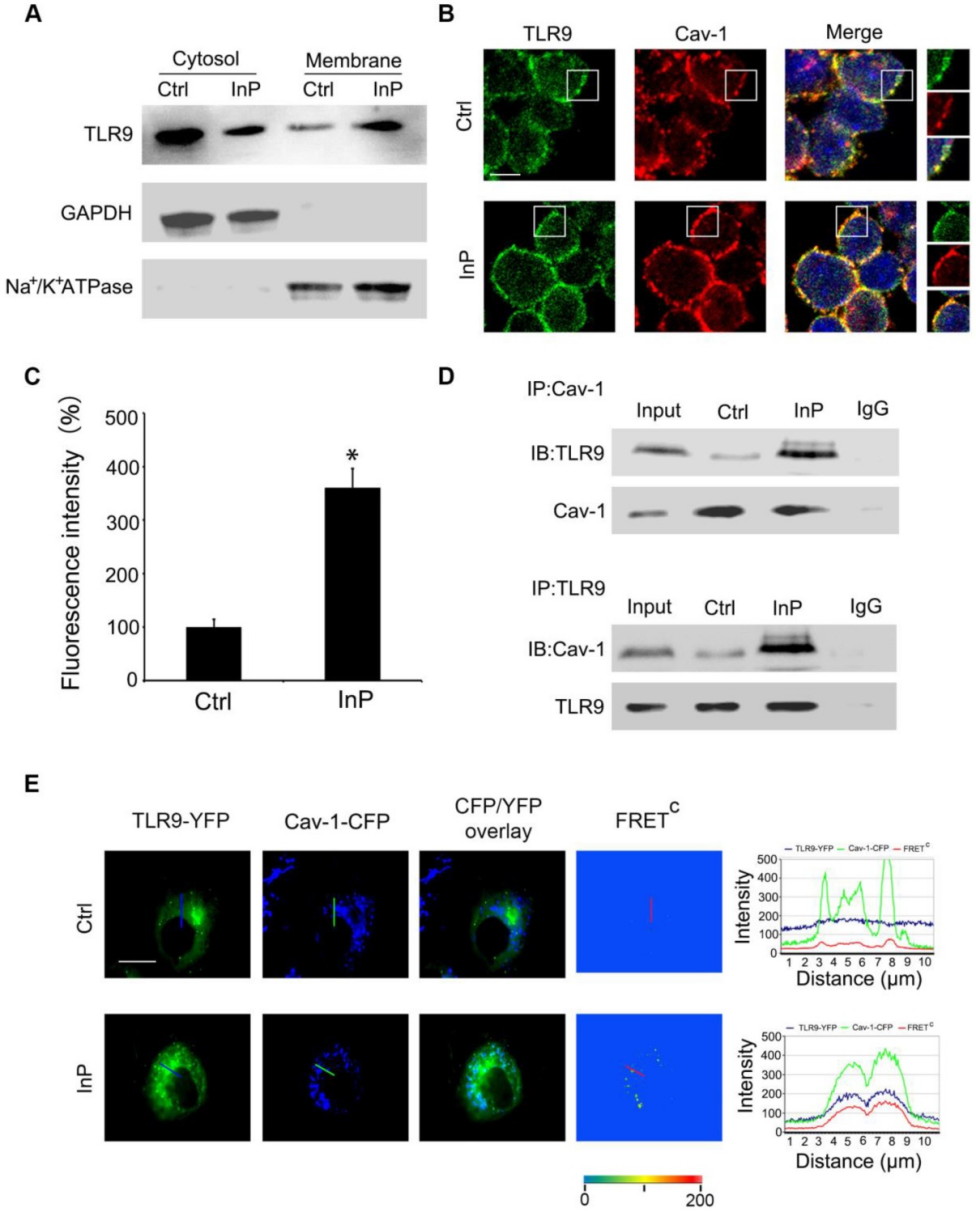
** Association of TLR9 with Cav-1 in MPLA mediated inflammatory preconditioning (InP). (A)** Western blot shows that InP promotes translocation of TLR9 from cytosol to membrane in HL60 cells. **(B)** Immunostaining of TLR9 and Cav-1 in HL60 cells stimulated with InP.** (C)** Fluorescence intensity of TLR9/Cav-1 colocalization on cell membrane. **(D)** Upper panel: Whole cell lysates from neutrophils treated with or without Co-IP were immunoprecipitated with anti-Cav-1 antibody, followed by immunoblotting with antibodies against TLR9 in mice peripheral blood neutrophils. IgG denotes immunoprecipitation with control IgG from the protein lysates of InP treated cells. Lower panel: The whole cell lysates from mice peripheral blood neutrophils treated with or without Co-IP were immunoprecipitated with anti-TLR9 antibody, followed by immunoblotting with antibody against Cav-1.** (E)** Representative images of TLR9-YFP, Cav-1-CFP and FRETC in H9c2 cells. InP led to FRET signals due to energy transfer from CFP of Cav-1 to YFP of TLR9. Fluorescence intensity measured along the line. 400×; n=20.

To determine how InP triggers TLR9 activation, we first assessed whether this process involved altered distribution of TLR9 within neutrophils. Given that TLRs translocation to the plasma membrane can occur under stimulated conditions 19-23**,** we hypothesized that a similar translocation event may happen with TLR9 in the setting of InP. To test this hypothesis, we first collected cytosol and plasma membrane fractions and measured the distribution of TLR9 in these fractions from HL60 cells. GAPDH and Na^+^/K^+^-ATPase were used as cytosol and membrane markers, respectively. As shown in Figure 3A, most of the TLR9 was distributed in the cytosol, with minimum expression on the cell membrane under control conditions, which is consistent with previous reports 24-26. However, InP triggered significant translocation of TLR9 from the cytosol to the plasma cell membrane in HL60 cells (Figure 3A).

Cav-1 has been shown to interact with various signaling molecules located in caveolae, which provide both spatial and temporal organization for cellular signal transduction 15. Given that TLR9 is translocated to the plasma membrane upon InP, we next sought to determine whether the activated TLR9 interacts with Cav-1. To do so, we first used MPLA to activate TLR9 in HL60 cells. In the control condition, co-localization of TLR9 and Cav-1 on the cell membrane was minimal (Figure 3B), and most of the TLR9 was in the cytosol, within the endoplastic reticulum (Figure S3A). After treatment with MPLA, a drastic increase in co-localization of TLR9 and Cav-1 on the cell membrane was observed (Figure 3C), with a corresponding decrease in TLR9 in the endoplastic reticulum (Figure S3B). We next used co-immunoprecipitation with an anti-Cav-1 or an anti-TLR9 antibody and tested the association between TLR9 and Cav-1 in the whole cell lysates of neutrophils extracted from InP-treated or non-treated mice. As expected, interactions between Cav-1 with TLR9 were minimal under control conditions (Figure 3D). In contrast, InP greatly enhanced the interaction of Cav-1 with TLR9 (Figure 3D). These results were further confirmed in HL60 cells and mice neutrophils when MPLA was used to activate TLR9* in vitro* (Figure S3C and S3D). We further measured the association of Cav-1 and TLR9 by using a fluorescence resonant energy transfer (FRET) assay. H9c2 cells, which have minimal expression of TLR9 and Cav-1, were transfected with Cav-1-CFP, TLR9-YFP, or both. Corrected FRET values (FRET^C^) were weak in all areas of cells without InP, suggesting that minimal amounts of TLR9 were in the membrane. InP treatment significantly increased the translocation of TLR9 to cell membrane microdomains and resulted in strong FRET fluorescence (Figure 3E). The signal intensity calculated along the line indicated strong FRET fluorescence across the plasma membrane under the InP condition. These results suggest that InP promotes the translocation of TLR9-YFP to the cell membrane and enhances the association between Cav-1-CFP and TLR9-YFP. Together, these results indicate that InP triggers the rapid association of membrane TLR9 with Cav-1 in neutrophils.

### The protective effect of mTLR9 in InP is Cav-1 dependent

**Figure 4:**
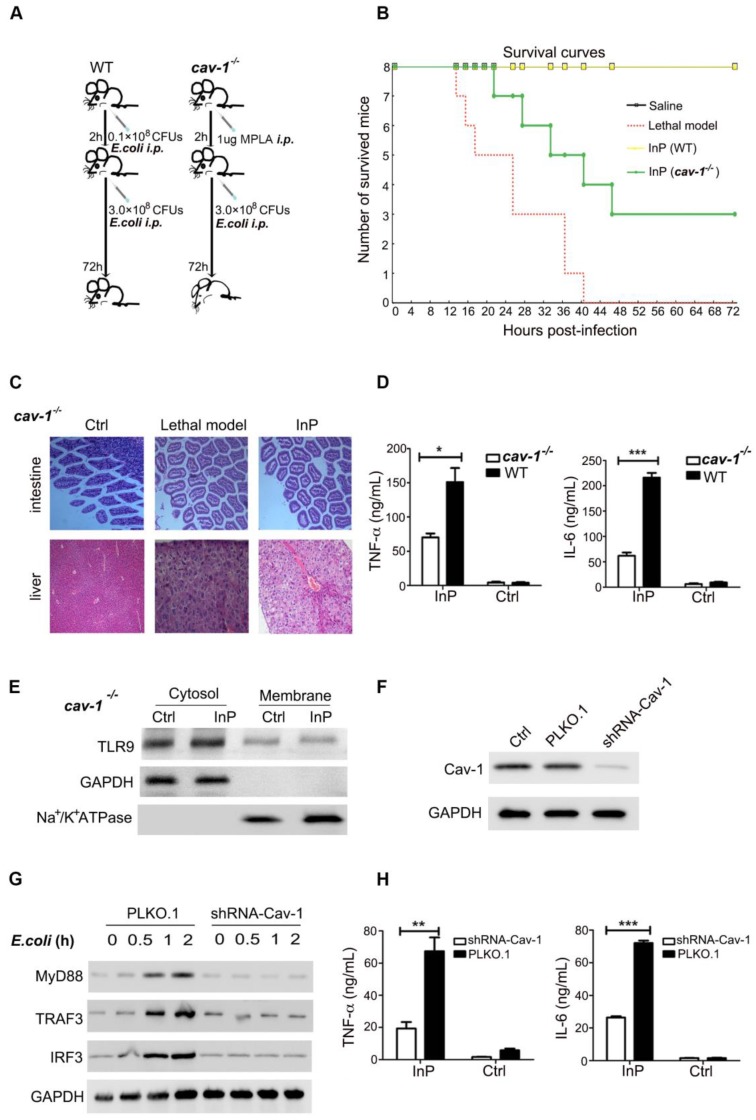
** The effect of MPLA mediated inflammatory preconditioning (InP) on *cav-1^-/-^* mice. (A)** Survival curves, n=8 mice per group. The mice were first given 0.1×10^8^ CFUs *E. coli*, followed by the lethal dose of *E. coli* (3×10^8^ CFUs) 2 h later,* *vs.* Saline. **(B)** Experimental procedures. (1) The WT mice were injected *i.p.* with 0.1×10^8^ CFUs* E. coli,* and 2 h later were injected with 3×10^8^ CFUs* E. coli* per mouse, and all 8 mice survived (InP). (2) C*av-1^-/^*^-^ mice were injected *i.p.* with 0.1×10^8^ *E. coli,* and 2 h later were injected with 3×10^8^ CFUs* E. coli,* and all 8 mice died. **(C)** Intestine and liver tissues of mice stained with H&E.** (D)** ELISA of TNF-α/IL-6 in the supernatants of peripheral blood neutrophils in mice stimulated with 0.1×10^8^ CFUs *E. coli* for 2 h. **P<0.01 (Student's *t* test). **(E)** Western blot shows that InP did not promote translocation of TLR9 from cytosol to cell membrane in *cav^-/-^* mice. **(F)** Western blot shows significant reduction of endogenous Cav-1 by shRNA against Cav-1 (Cav-1 shRNA). **(G)** Immunoblot of TLR9, MyD88, TRAF3 and IRF3 in HL60 cells transfected Cav-1 shRNA and then stimulated with 0.1×10^8^ CFUs *E. coli* for indicated times. Similar results were obtained in three independent experiments. **(H)** ELISA of TNF-α/IL-6 in the supernatants of HL60 cells transfected Cav-1 shRNA, stimulated with 0.1×10^8^ CFUs *E. coli* for 2 h. **P<0.01 (Student's *t* test).

Because InP promotes the translocation of TLR9 to the cell membrane and because TLR9 is associated with Cav-1, we reasoned that Cav-1 is likely to be essential for the TLR9-mediated protective effect during sepsis. Confirming our theory, we found that all WT mice with InP survived an *E. coli* attack, but the protective effect of InP was largely blocked in *cav-1*^-/-^ mice, resulting in the death of half of the animals (Figure 4A & 4B). Severe liver and intestinal damage was also observed in the *cav-1*^-/-^ mice even under InP conditions (Figure 4C). Further, activation of TLR-9 downstream pathways, including TNF-α and IL-6, was largely inhibited in the *cav-1*^-/-^ mice compared with the WT mice (Figure 4D).

To evaluate whether Cav-1 is critical for the activation of downstream effectors of TLR9, we used shRNA to knock down Cav-1 expression in HL60 cells and found that of Cav-1 KD drastically reduced the translocation of TLR-9 from the cytosol to the plasma membrane (Figure 4E & 4F). In line with the findings from the *cav*-1^-/-^ mice, Cav-1 KD markedly inhibited *E. coli* and CpG-induced MyD88, TRAF3 and IRF3 expression (Figure 4G & Figure S4A). Meanwhile, production of both TNF-α and IL-6 was significantly blocked in the Cav-1 KD group (Figure 4H, S4B & S4C). These results demonstrated that Cav-1 was required for TLR9-mediated MyD88-TRAF3-IRF3 signaling pathway activation in neutrophils in the setting of InP.

### TLR9 binds to Cav-1 through its caveolin-binding motif sequence, and the caveolin-scaffolding domain of Cav-1 is responsible for this interaction

**Figure 5:**
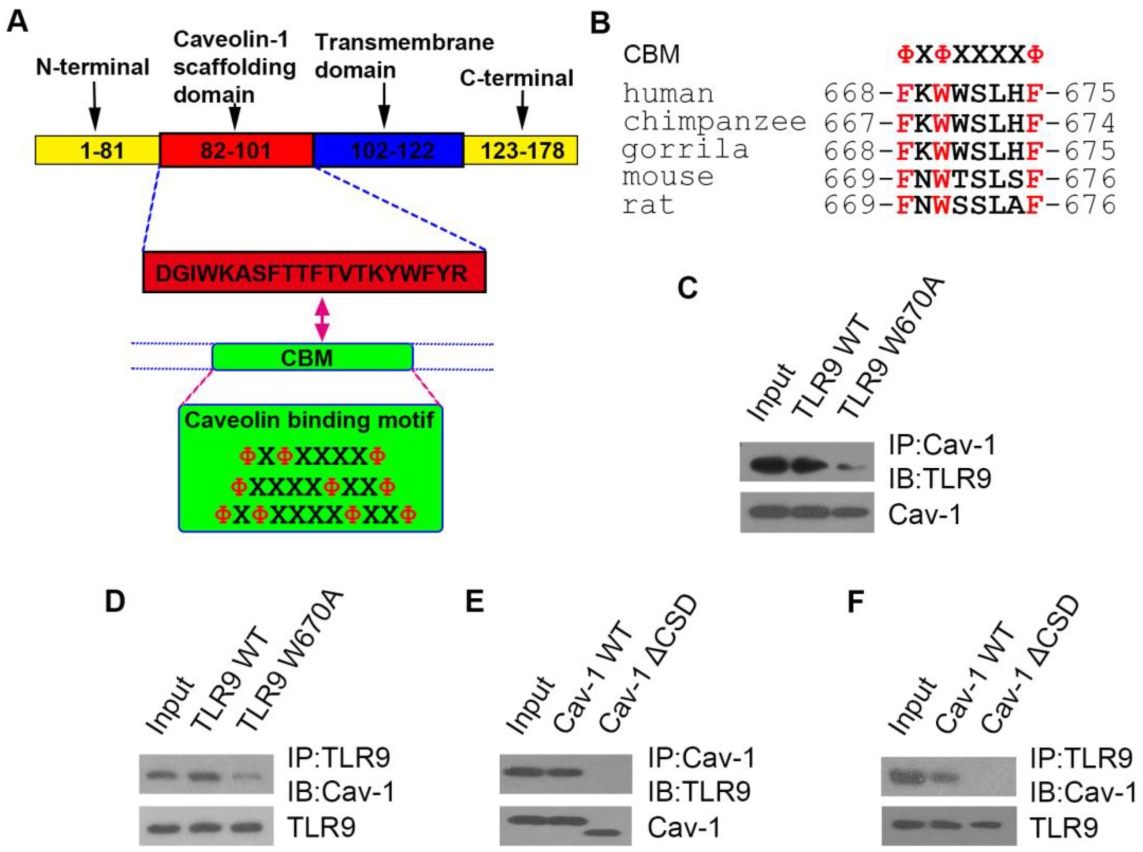
** The interaction sites of TLR9 with Cav-1. (A)** Schematic representation of the domains of Cav-1 and the amino acid sequences of the caveolin binding motif. X: any amino acid; Φ: aromatic amino acid. **(B)** Possible caveolin binding motif in different species. **(C, D)** H9c2 cells were co-transfected with Cav-1 and TLR9 WT (or TLR9 W670A), and 24h post-transfection, cell lysates were subjected to immunoprecipitation with anti-Cav-1 (C) or anti-TLR9 (D) antibodies. The immunoprecipitants were then detected with the indicated antibodies. **(E, F)** H9c2 cells were co-transfected with TLR9 and Cav-1 WT (or Cav-1 ΔCSD), and 24 h later cell lysates were subjected to immunoprecipitation with anti-Cav-1 (E) or anti-TLR9 (F) antibodies. The immunoprecipitants were then detected with the indicated antibodies.

Thus far, we showed that TLR9 and Cav-1 interaction is critical for the protective effect of InP against sepsis, but it was unclear how these molecules interact with one another. Cav-1 is known to interact with most of its target proteins via the canonic caveolin binding motif (CBM), ΦXΦXXXXΦ, ΦXXXXΦXXΦ or ΦXΦXXXXΦXXΦ, where Φ is an aromatic amino acid (Y, W, and F) and X is any amino acid (Figure 5A). Human TLR9 contains a potential CBM sequence (668-FXWXSLXF-675), which is highly conserved in both primate and rodent species (Figure 5B). To determine whether TLR9 interacts with Cav-1 through this CBM sequence, we mutated the aromatic amino acid W670 to the non-aromatic amino acid alanine (A). As expected, the aromatic to non-aromatic amino acid mutation greatly abolished the interaction between Cav-1 and TLR9 (Figure 5C & 5D). We further generated another mutant in which the caveolin-scaffolding domain (CSD) was deleted. The CSD deletion mutant could not interact with TLR9 (Figure 5E & 5F). These results suggest that TLR9 binds to Cav-1 through its CBM sequence (668-FXWXSLXF-675) and the CSD of Cav-1.

### TLR9-Cav-1 signaling activation is associated with survival of patients with sepsis

**Figure 6:**
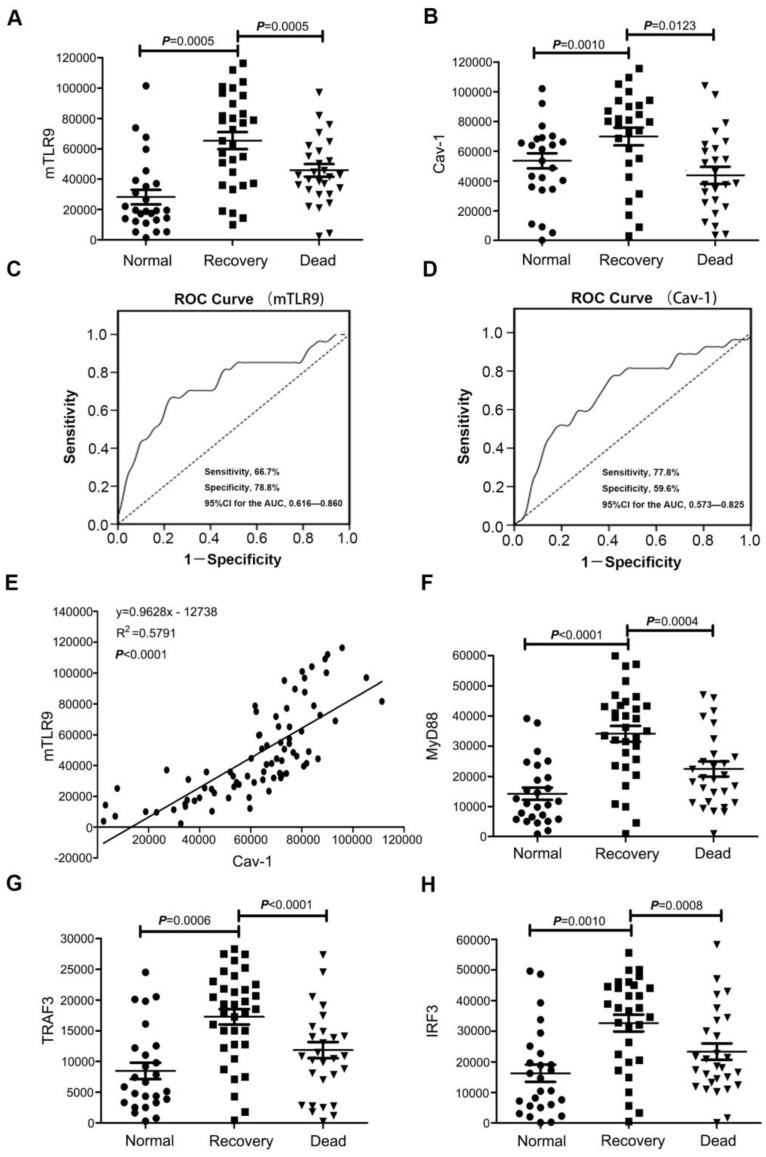
** Expression of TLR9-Cav-1 signaling proteins in the neutrophils of patients with sepsis. (A)** Membrane TLR9 expression.** (B)** Cav-1 expression.** (C)** ROC curve for mTLR9. *P*<0.05. **(D)** ROC curve for Cav-1. *P*<0.05. **(E)** Association of Cav-1 with surface TLR9 expression in neutrophils. r2 =0.5791. **(F)** MyD88 expression.** (G)** TRAF3 expression. **(H)** IRF3 expression.

To confirm that TLR9-Cav-1 mediated sepsis protection is also translatable to patients with bacterial sepsis, we collected blood samples from patients with sepsis to characterize the protective role of TLR9-Cav-1. A total of 60 sepsis patients were selected, 30 of whom eventually died from sepsis (Dead Group) and 30 survived (Recovery Group). All blood samples were drawn before therapy, and neutrophils were separated immediately. An additional 30 age-matched healthy subjects were chosen as well. Demographics and disease-related information for these patients are listed in Supplemental Table S2. Membrane TLR9 expression was found to be significantly elevated among sepsis patients compared with normal donors (Figure 6A). Further stratification showed that TLR9 expression in the Recovery group was significantly higher than that in the Dead group (*p*=0.0005), and the normal donor group had the lowest TLR9 expression. Cav-1 expression was also higher in the Recovery group than in the Dead group (Figure 6B).

The areas under the empiric receiver-operating-characteristic curves for membrane TLR9/Cav-1 were 0.738 (*p*<0.05, Figure 6C) and 0.699 (*p*<0.05, Figure 6D), respectively. The sensitivity, specificity, accuracy, cutoff, and 95%CI for the AUC analyses are presented in supplemental table 3. Membrane TLR9 was also found to correlate with that of Cav-1 (r^2^=0.5791) (Figure 6E). Flow cytometry analysis further revealed that downstream effectors of TLR9, including IRF3, MyD88, and TRAF3, were elevated in all of the sepsis patients compared with the normal donors (Figure 6F, 6G & 6H), and was correlated with improved prognosis in patients with sepsis. Evaluation of other commonly used diagnostic biomarkers including white blood cells (WBC), C-reactive protein (CRP) and procalcitonin (PCT) showed no statistical differences between the Recovery and Dead groups (Figure S5). Therefore, our results indicated that membrane TLR9-Cav-1 is activated in the setting of bacterial sepsis, and that upregulation was correlated with improved clinical outcomes among patients with sepsis.

## Discussion

Although neutrophils have a key role in inflammation and infection, the details of the mechanisms underlying neutrophil-mediated protection in sepsis are not completely understood. Here, we defined a novel protective process, the InP, in which MPLA protects the organism against subsequent fatal doses of bacteria. In this study, we provide novel molecular insights into the essential role of TLR9 activation in the neutrophil-dependent protection from sepsis. Specifically, TLR9 constitutively associates with Cav-1 and facilitates MyD88-mediated TRAF3 and IRF3 signal transduction, providing the observed InP effect in fatal doses of bacterial infection (Figure S6).

In our previous work, we set up the lethal *E. coli* mouse model 8. From that model, we constructed the protective InP mouse model, which simulates the experimental procedure of ischemic preconditioning, in which short periods of ischemia can protect the heart from a subsequent ischemic insult 27-30. Similar to ischemic preconditioning, we noted that InP had a protective effect in a short time window, with the maximum effect occurring 2 h before the lethal dose. This suggests that the InP effect may not be related to the mRNA transcription process. However, during the InP process, we observed increased TLR9 and Cav-1 mRNA expression. This is comparable to the second time-window of ischemic preconditioning, during which the mRNA transcription is elevated. Since this process began at 24 hours and lasted up to 72 hours, we also evaluated the inP at 24, 48, and 72 h (Figure 1A). To our surprise, unlike ischemic preconditioning, we did not observe an obvious InP effect in these groups, even though the mRNAs detected in these groups were elevated. Additional experiments are required to reveal the molecular mechanism underlying these differences.

We also conducted InP induction experiments with *E. coli* genomic DNA, plasmid, and LPS. The survival rates revealed that genomic DNA and plasmid had no protective effect. A low dose of LPS could prolong the survival period, but it also showed noticeable toxic effects. Published studies from other groups have also presented similar observations 31, 32. For instance, sublethal doses of LPS induce endotoxin tolerance, a temporary state of hyporesponsiveness of the innate immune system, which renders mice resistant to a subsequent lethal LPS challenge. However, the survival mice exhibited toxic symptoms, such as body temperature decrease, fur roughness, loose stools, body weight drop, and less mobility. The specific mechanisms need further exploration.

The fact that MPLA-mediated mTLR9^+^ neutrophil recruitment depends on a unique pattern of adhesion receptors could pave the way for novel strategies to treat inflammation. Many types of cells are present in peritoneal lavage fluid 33-35, but most are neutrophils. Neutrophils stimulated by MPLA will become mTLR9^+^ neutrophils (mTLR9^low^ or mTLR9^high^), and the effect of these two cell subtypes is a key mechanism of InP. So why do neutrophils have these two cell types? Is the cause of the stimulus time or cell type? Could these two types of cells be transformed into each other? If mTLR9^low^ could be converted to mTLR9^high^, that would mean that the time point of the stimulus is the key factor for this conversion and that the conversion may be consistent with the role of Cav-1 internalization 36-40. If mTLR9^low^ cannot be converted to mTLR9^high^, then neutrophil cell type could be the cause, and mTLR9 may serve as a protein marker of neutrophil subtype. Research on the cell membrane protein Cav-1 shows that not all neutrophils express Cav-1^high^, and that neutrophils can be phenotyped according to the different expression of membrane proteins 41-43. Our experimental results showed that TLR9 could bind with Cav-1 on the cell membrane, and Cav-1 may be internalized into the cytoplasm from the cell membrane. The internalization of Cav-1 may be sufficient for the next step, the translocation of TLR9 from the cytoplasm to the cell membrane.

Currently, WBC, CRP, and PCT are commonly used indicators of sepsis in clinical settings 44, 45. Although they are useful for the diagnosis of sepsis, they cannot predict the outcome of therapy, as we found no statistical differences in their levels between the Recovery and Dead groups. Biomarkers that can distinguish therapeutic outcomes in sepsis, so that the patients can be provided the appropriate treatment, are urgently required. Our finding suggests that TLR9/Cav-1 signaling might be useful for predicting therapeutic outcomes in patients with sepsis. Because the average hospitalization time for patients with sepsis is about 3 weeks, we further divided the Recovery group into two subgroups: good recovery, *i.e.*, hospitalized for less than 3 weeks, and bad recovery,* i.e.*, hospitalized for 3 weeks or more. Interestingly, although these subgroups did not differ in WBC, CRP or PCT values, the good recovery subgroup had relatively higher expression levels of TLR9 and Cav-1 than the bad recovery subgroup (data not shown). Meanwhile, downstream of TLR9, MyD88, TRAF3, and IRF3 were also higher in the good recovery subgroup. Collectively, these findings suggest that TLR9-Cav-1 signaling could be a powerful indicator of therapeutic outcome in patients with sepsis (Figure 6).

In summary, our findings indicated that MPLA mediated InP could promote the rapid translocation and caveolar targeting of membrane TLR9. We found the TLR9-Cav-1 axis to be a key signaling pathway in the regulation of neutrophil-dependent InP. This research suggests that mTLR9^+^ neutrophils could be an attractive tool for studying new protective pathways for the diagnosis and therapy of sepsis.

## Methods

### Antibodies and Reagents

Mouse or goat IgG directed against TLR9 or Cav-1 and rabbit IgG directed against TLR9, Cav-1, GAPDH, TLR4, IRAK-4, TRAF6, TRAF3, MyD88, IRF7, IRF3, p38MAPK and JNK were obtained from Abcam (Abcam, USA). Anti-Ly6G and anti-CD11b were from Abcam Plc (Cambridge, UK). Mouse TNF-α (RAB0477) and IL-6 (RAB0308) ELISA Kits were purchased from Sigma** (**Sigma-Aldrich China, Shanghai). MyD88 homodimerization inhibitor peptide (NBP2-29328) was purchased from Novus Biologicals (Novus, USA).

MPLA (L 5399) was from Sigma (Sigma, USA) TLR9-specific shRNA (sc-40270-SH), Cav-1 shRNA (sc-29241-SH) and transfection reagent (sc-108061) were bought from Santa Cruz Biotechnology (Santa Cruz, CA). Anti-TLR9 for flow cytometry (560425, BD), Anti-Cav-1 for Flow Cytometry (PA1-064, Invitrogen), anti-MyD88 for flow cytometry (566354, BD), anti-IRF3 for flow cytometry (566347, BD), anti-TRAF3 for flow cytometry (PA5-29091, Invitrogen) and Fugene HD transfection reagents were obtained from Promega (Promega, USA). The full-length Cav-1 was ligated into pECFP-C1 and TLR9 was inserted into pEYFP-N1. Anti-mouse-IgG3-FITC was from Southern Biotech (Birmingham, USA); anti-human IgG-FITC, anti-rat Alexa568, and anti-rabbit Alexa555 were purchased from Thermo Fisher Scientific (Braunschweig, Germany); anti-FITC-Alexa488 was from Life Technologies (Darmstadt, Germany); and DAPI was from Sigma (Deisenhofen, Germany).

### *E. coli* Strain and Mice

*E. coli* strain JM109 was recovered from lyophilized powder by resuspending in LB medium and culturing overnight on an LB agar plate at 37°C. Single colonies of *E. coli* on the plate were used as a group of original seed *E. coli*.

*tlr9*^-/-^ and *cav-1*^-/-^ mice were purchased from Taconic Biosciences (Hudson, USA). ICR mice were 6-8 weeks old, weighed 20 ± 2 g, and were obtained from Jilin University Animal Center (Chang Chun, China). They were fed in the laboratory for 1 week before the experiment. Mice were kept in standard pathogen-free and temperature-controlled units. Food and water were supplied *ad libitum*. The animal protocol was approved by the Scientific Investigation Board of Science and Technology (Jilin).

### Preparation of *E. coli* Culture

To prepare the *E. coli* culture, a single colony of *E. coli* was picked up and cultured in 5 mL LB medium (37°C, 200 rpm). When the OD value (A600) of the medium reached 0.7, the *E. coli* cells were harvested from the culture by centrifugation and mixed with 20% glycerol. The resultant glycerol *E. coli* was stored at -20°C as working *E. coli* seeds. The working seed *E. coli* was cultured into LB solution and incubated at 37°C with agitation until the OD value reached 0.7. At this point, the cells were harvested by centrifugation (4,000 X g, 5 min) and an *E. coli* pellet was obtained. The *E. coli* pellet was serially diluted tenfold, plated on an eosin methylene blue agar plate, and cultured at 37°C for 14 hours. The colony-forming units (CFU) of the *E. coli* culture were calculated and determined to be approximately 0.8 × 10^8^ CFU. For experiments involving mice, the *E. coli* pellet was washed twice with sterile 0.9% NaCl (saline) and then resuspended in 0.1 mL saline containing 0.1×10^8^ CFU and 1 mL saline containing 3 × 10^8^ CFU of *E. coli*, respectively. Those solutions were then ready to be used to infect the mice 8.

### Induction of Bacterial Inflammation Lethal Model and Inflammatory Preconditioning Model

Mice were randomly separated into three groups: saline control group, inflammation lethal model group, and the inflammatory preconditioning (InP) group. The saline control group received *i.p* injections of 1 mL saline. The inflammation lethal model group received *i.p.* injections of 1 mL *E. coli* (3×10^8^ CFU). The inflammation preconditioning (InP) group received *i.p.* injections of 0.1 mL *E. coli* (0.1×10^8^ CFU), and 2 hours later were given *i.p.* injections of 1 mL *E. coli* (3×10^8^ CFU). MPLA-InP group received *i.p.* injections of 0.1 mL MPLA (0.01 μg/μL), and 2 hours later were given *i.p.* injections of 1 mL *E. coli* (3×10^8^ CFU). Mouse survival was recorded six times daily for up to three days (72 h).

### Cell Culture and Transfection and RNA Interference Procedures

HL60 cells were cultured in DMEM/F12 containing 10% fetal bovine serum, 2 mM L-glutamine, and antibiotics (penicillin/streptomycin). HL60 cells were transfected with cDNAs using Fugene HD transfection reagent according to the manufacturer's instructions. HL60 cells were stably transfected with TLR9/Cav-1-specific shRNA. At 48 hours after transfection, western blotting as used to verify knockdown of the targeted proteins.

### Peripheral Blood Neutrophil Preparations

Briefly, peripheral samples were withdrawn from the mice and placed in EDTA tubes and mixed with sample diluent at a ratio of 1:1, to reach a final volume of 20 mL. Neutrophils were isolated with a separation kit. To form a liquid gradient, we added reagents A and C to the glass tubes in a ratio of 3:2. Then, the blood sample was added to the tubes gently, gradient centrifuged at 500 x *g* for 30 minutes. The second milky cell layer was the isolated neutrophils. The neutrophils were washed 2 times in cleaning fluid, centrifuged at 550 x *g* for 5 min. The isolated neutrophils were re-suspended in RPMI1640 medium supplemented with 10 % fetal bovine serum, 2 mM L-glutamine, and 100 U/100 μg/mL of penicillin/streptomycin.

### Flow Cytometry to Detect of Percentages of Cell Types and Cell Sorting

Percentages of peritoneal lavage cells were analyzed by flow cytometry with cell membrane marker proteins characteristic of neutrophils, B cells, T cells, macrophages, and NK cells. Lavage cells were collected in a non-enzymatic cell dissociation solution and concentrated by centrifugation. Cell pellets (5 × 10^5^ cells) were rinsed twice with 500 μL of cold staining solution (1X PBS with 0.1% sodium azide). Cell membrane markers were measured by using the corresponding antibodies by incubation at 4ºC for 45 min. All samples were analyzed by a Coulter Epics XL flow cytometer (Beckman Coulter, High Wycombe, UK). Data were analyzed by Expo 32 software (Beckman Coulter). At least 10,000 cells were analyzed for each group. Isotype controls were used for each sample, and the percentage of positive staining in each sample was calculated.

Peritoneal lavage cells were harvested from mice (n≥ 6 per group) that had been either infected with 0.1×10^8^ CFU *E. coli* or injected with saline. The peritoneal lavage cells were incubated with a fluorescence-conjugated antibody against Ly6G for 30 minutes at 4ºC and washed three times with cold PBS. The neutrophils were sorted by selection for Ly6G^+^ cells, and the non-neutrophils were sorted by selection for Ly6G^-^ cells, according to the manufacturer's recommendations. Neutrophils (defined as Ly6G^+^ cells with >90% purity of living cells) and non-neutrophils were obtained. ICR mice were infected with 0.1×10^8^ CFU *E. coli* or injected with saline 2 hours before their peritoneal lavage cells were harvested. The cells were incubated with fluorescence-labeled antibody to Ly6G, followed by detection with flow cytometry.

### Flow Cytometry to Detect of Blood Samples from Patients with Sepsis

Freshly collected EDTA-anti coagulated whole blood was incubated from specimens collected upon admission. Cells were treated with 1 × FACS Lysing Solution (BD Biosciences, CA) for 10 min at room temperature. Cells were washed, aliquoted into 12 × 75 mm polystyrene tubes (Falcon), and incubated for 30 min at room temperature with membrane staining antibody. After washing, cells were treated with 250 μL BD Fixation/Permeabilization solution for 20 minutes at room temperature. After cells were washed two times in 1×BD Perm/Wash buffer, cells were stained for flow cytometry, typically with anti-TLR9/MyD88/Cav-1/TRAF3/IRF3. Cells were then washed two more times and resuspended in staining buffer before flow cytometric analysis.

### Quantitative Real Time Reverse Transcription PCR (qRT-PCR) Analysis

Total RNAs were harvested by using the Trizol reagent per the manufacturer's instructions (Invitrogen). Reverse transcription of equal amounts of RNA was done with a first-strand cDNA synthesis kit (Invitrogen) with random hexamers as primers. The qRT-PCR experiment was done with an ABI 7500 Real Time PCR System (Applied Biosystems, Carlsbad, CA). Each cDNA sample of each target gene (TLR9, TLR4, MyD88, IRAK4, TRAF3, TRAF6, P38, JNK, IRF3, IRF7, TNF-α) was detected in triplicate and results were normalized by GAPDH. All primer designs were obtained by Primer Express software (Applied Biosystems, Carlsbad, CA) and purchased from Sangon Biotech (Sangon, Shanghai). The primers used for the amplification reactions are listed in Supplemental Table S1.

### Western Blotting

Western blotting was done using standard protocol. Briefly, the samples were lysed and denatured in 5× sample buffer. Same amounts of proteins were separated on a 10% SDS-polyacrylamide gel, and transferred onto a nitrocellulose membrane. The nitrocellulose membrane was incubated with 5% non-fat milk in Tris-buffered saline (150 mM NaCl, 20 mM Tris-HCl, pH7.4) with primary antibody overnight at 4°C. After washing, the membrane was further incubated with secondary antibody for 45 minutes and proteins were detected with an ECL detection system. Image J software was used to measure the band intensity 15.

### Co-Immunoprecipitation

Immunoprecipitation studies were done as described previously 9. Peritoneal lavage cells were isolated, homogenized, lysed, and centrifuged, and the supernatant was collected afterward. After a 2-hour incubation at 4°C, the complex containing supernatant and antibody against Cav-1 or TLR9 was mixed with r-protein-G agarose. The beads were washed four times with solubilization buffer to remove the bound proteins and heated at 95°C for 5 min in SDS sample buffer. Samples were separated by SDS-polyacrylamide gels and then transferred onto nitrocellulose membranes, blocked with 5% milk, incubated with primary antibodies and secondary antibodies, then measured with an ECL detection system.

### Measurement of Fluorescence Resonance Energy Transfer (FRET)

24 h before the experiment, H9c2 cells were first transfected with TLR9-YFP or/and Cav-1-CFP plasmids. Images were taken sequentially under CFP, YFP and FRET filter channels as we mentioned before. The donor filter was set as CFP, and acceptor was YFP. In each image, an area with no cells was chosen to measure the background value. This number was deducted from the original images before performing FRET analysis. Corrected FRET (FRET^C^) was calculated as follows: FRET^C^ = FRET - (0.5 × CFP) - (0.5 × YFP), where FRET, CFP and YFP represents cell fluorescence obtained under FRET, CFP, and YFP channels with background subtracted. The 0.5 values were the percentage of bleed-through of either CFP or YFP channels, calculated from images with either CFP or YFP fluorescence. All analyses were done with MetaMorph. FRET^C^ images were shown in pseudocolor 15.

### Statistical Analysis

All experiments were performed three times and the results reported as mean ± standard error (SE). Statistical differences among multiple groups were analyzed by ANOVA. Statistical significance was defined as *p< 0.05* (*) and *p<0.01* (**).

## Supplementary Material

Supplementary figures and tables.
