# Supplementary material for: Membrane TLR9 Positive Neutrophil Mediated MPLA Protects Against Fatal Bacterial Sepsis
Source: Theranostics. 2019 Aug 14;9(21):6269–83. doi: 10.7150/thno.37139 (PMC6735515; doi:10.7150/thno.37139)
Supplement: Supplementary file 1 — Supplementary figures and tables. [file thnov09p6269s1.pdf]

Supplemental Figures and Legends

Figure S1. The protective effect of peritoneal lavage fluid in MPLA mediated inflammatory preconditioning (InP).

Figure S2. The effect of TLR9 in CpG ODN-induced inflammatory preconditioning (InP).

Figure S3. The effect of MPLA mediated inflammatory preconditioning (InP) on TLR9 translocation and association of TLR9 with Cav-1.

Figure S4. The effect of Cav-1 in CpG ODN-induced inflammatory preconditioning (InP).

Figure S5. Biomarkers commonly used for the clinical diagnosis of sepsis.

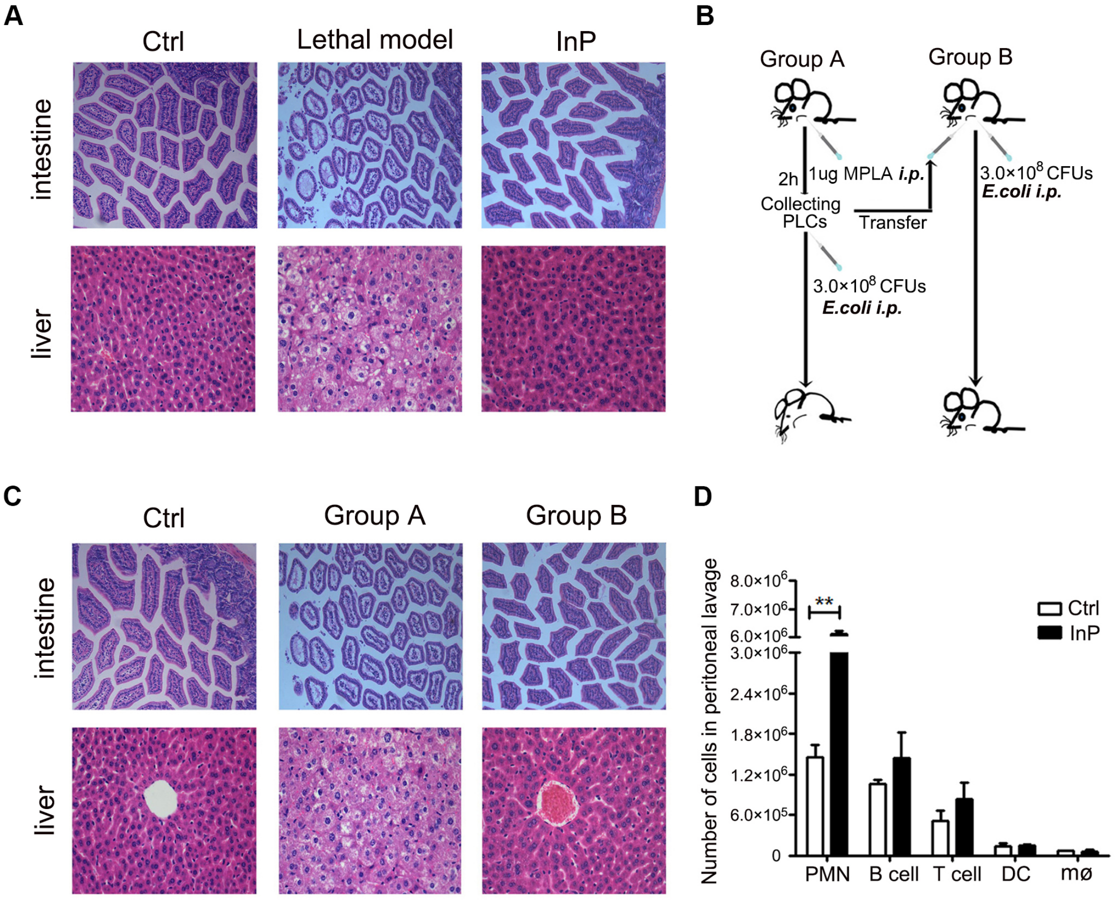

Figure S1. The protective effect of peritoneal lavage fluid in MPLA mediated inflammatory preconditioning (InP). **(A)** Intestine and liver tissues of mice with InP stained with H&E. **(B)** Experimental procedure for the transfer of peritoneal lavage fluid. **(C)** Intestine and liver tissues of mice staining with H&E for the peritoneal lavage transfer experiment. **(D)** The numbers of peritoneal lavage cells, InP vs. saline.

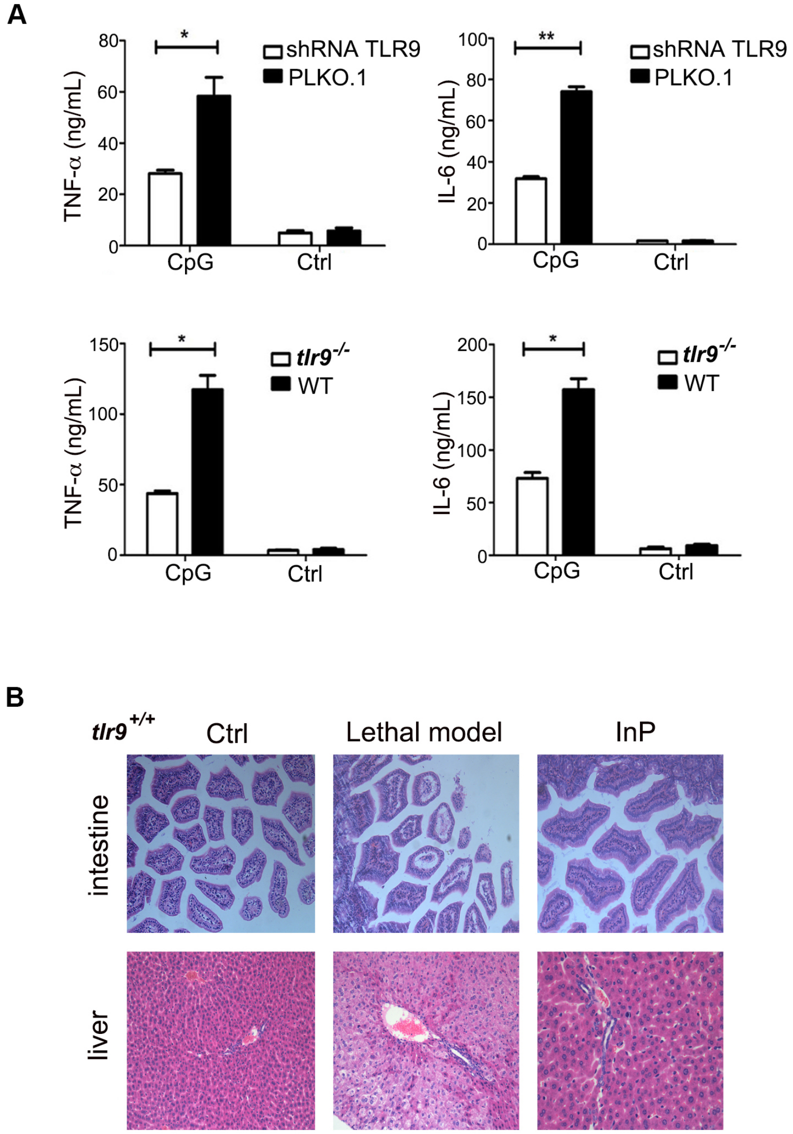

Figure S2. The effect of TLR9 in CpG ODN-induced inflammatory preconditioning (InP). (A) ELISA of TNF-α/IL-6 in the supernatants of HL60 cells stably transfected with TLR9 shRNA and PLKO.1 (control vector), stimulated with 10 mg/mL CpG 2006 for 2 h. ELISA of TNF-α/IL-6 in the supernatants of peripheral blood neutrophils in *tlr9*$^{-/-}$ mice stimulated with 10 mg/mL CpG 1826 for 2 h. **P*<0.05, ***P*<0.01 (Student's *t* test). **(B)** Intestine and liver tissues of *tlr9*$^{+/+}$ mice stained with H&E.

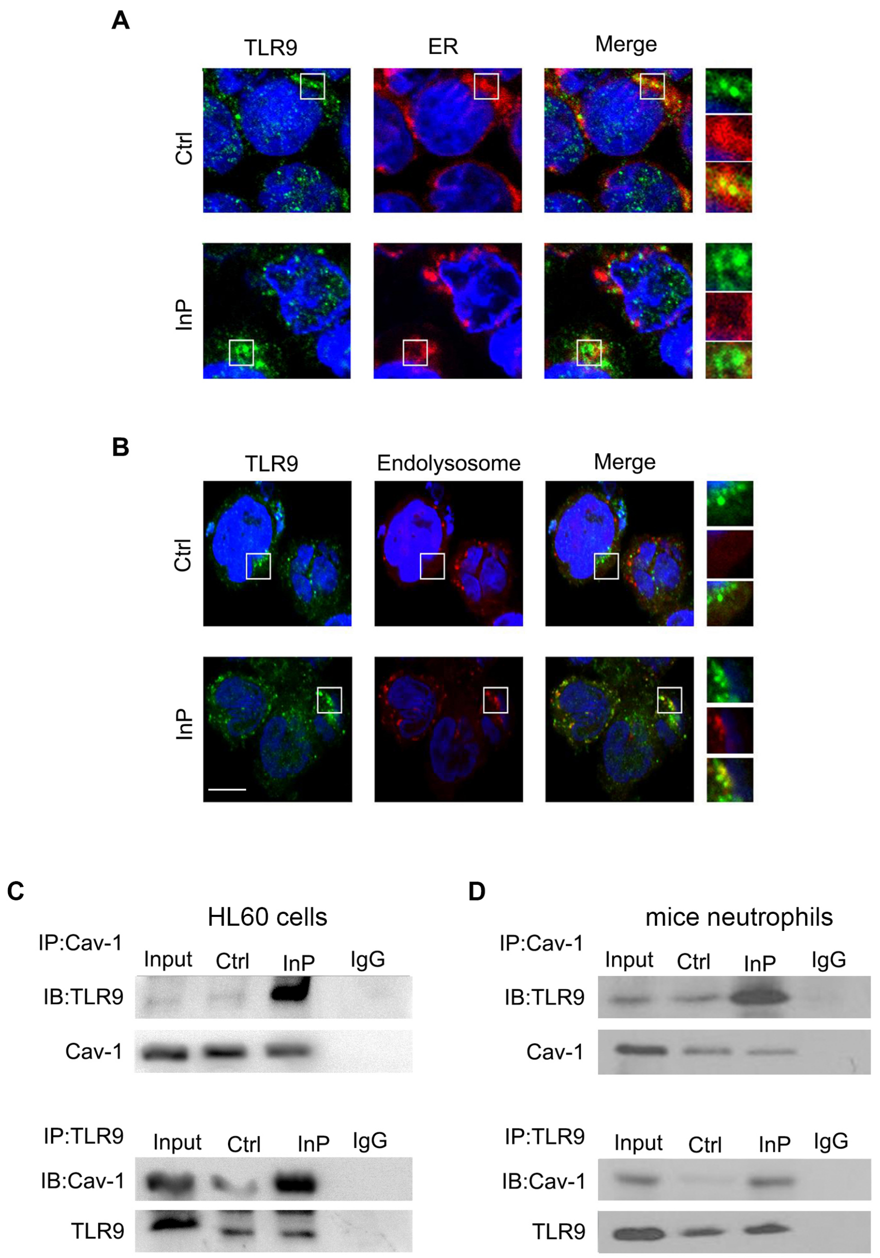

Figure S3. The effect of MPLA mediated inflammatory preconditioning (InP) on TLR9 translocation and association of TLR9 with Cav-1. (A)(B) The effect of InP on translocation of TLR9 in HL60 cells. TLR9 is in the endoplasmic reticulum (ER) under normal conditions, and TLR9 translocates from the ER to endolysosome in cells treated with *E. coli* for 2 h. **(C)** Effect of InP on co-precipitation of Cav-1 and TLR9 in HL60 cells. The caveolin-rich fractions from HL60 cells treated or not treated with Co-IP were immunoprecipitated with anti-Cav-1 antibody, followed by immunoblotting with antibodies against TLR9. The caveolin-rich fractions from HL60 cells treated or not treated with Co-IP were immunoprecipitated with anti-TLR9 antibody, followed by immunoblotting with antibodies against Cav-1. **(D)** Effect of InP on co-precipitation of Cav-1 and TLR9 in mice neutrophils. (n=8)

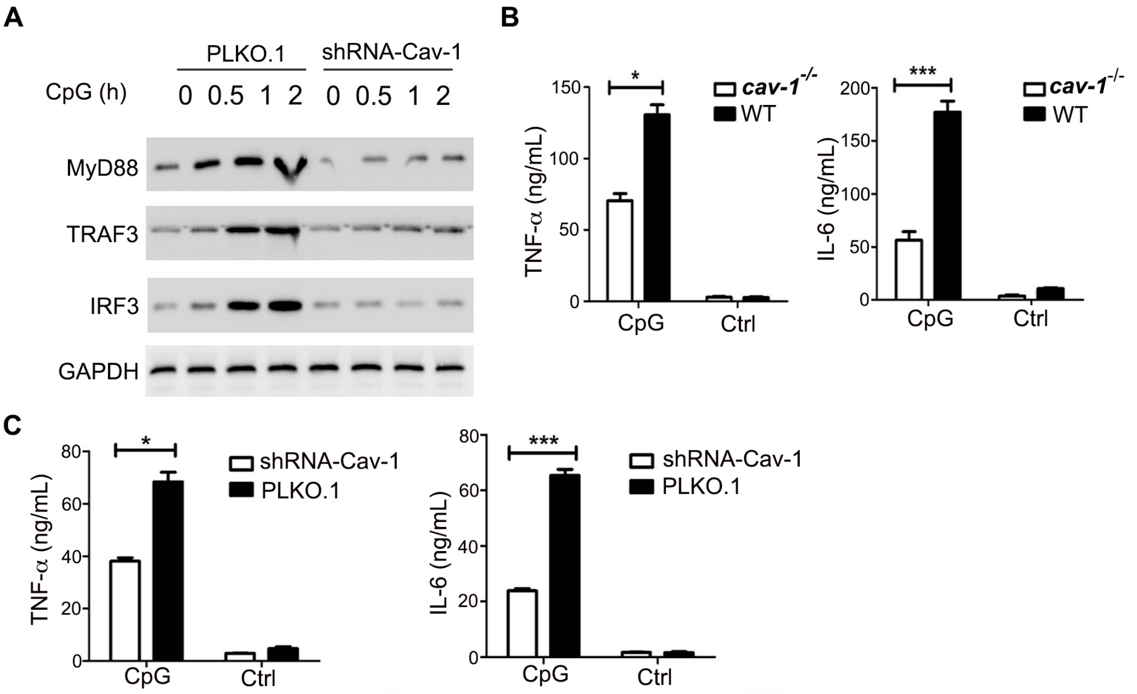

Figure S4. The effect of Cav-1 in CpG ODN-induced inflammatory preconditioning (InP). (A) Immunoblot of TLR9, MyD88, TRAF3, and IRF3 in HL60 cells transfected with Cav-1 shRNA and then stimulated with 10 mg/mL CpG 2006 for the indicated times. Similar results were obtained in three independent experiments. **(B)** ELISA of TNF-α/IL-6 in the supernatants of peripheral blood neutrophils in *cav-1*$^{-/-}$ mice stimulated with 10 mg/mL CpG 1826 for 2 h. **(C)** ELISA of TNF-α/IL-6 in the supernatants of HL60 cells transfected Cav-1 shRNA, stimulated with 10 mg/mL CpG 2006 for 2 h. ***P*<0.01 (Student's *t* test).

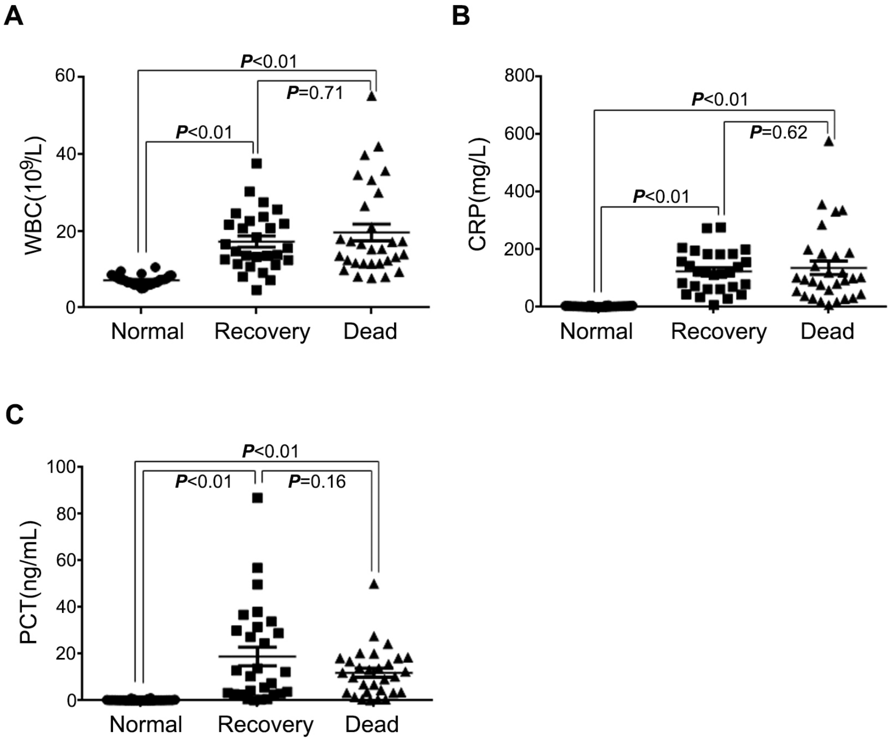

Figure S5. Biomarkers commonly used for the clinical diagnosis of sepsis. (A) White blood cell (WBC) counts in normal donors (Normal), patients who recovered from sepsis (Recovery) and patients who died of sepsis (Dead). **(B)** Blood C-reactive protein (CRP) levels in the above three groups. **(C)** Blood procalcitonin (PCT) levels in the above three groups.

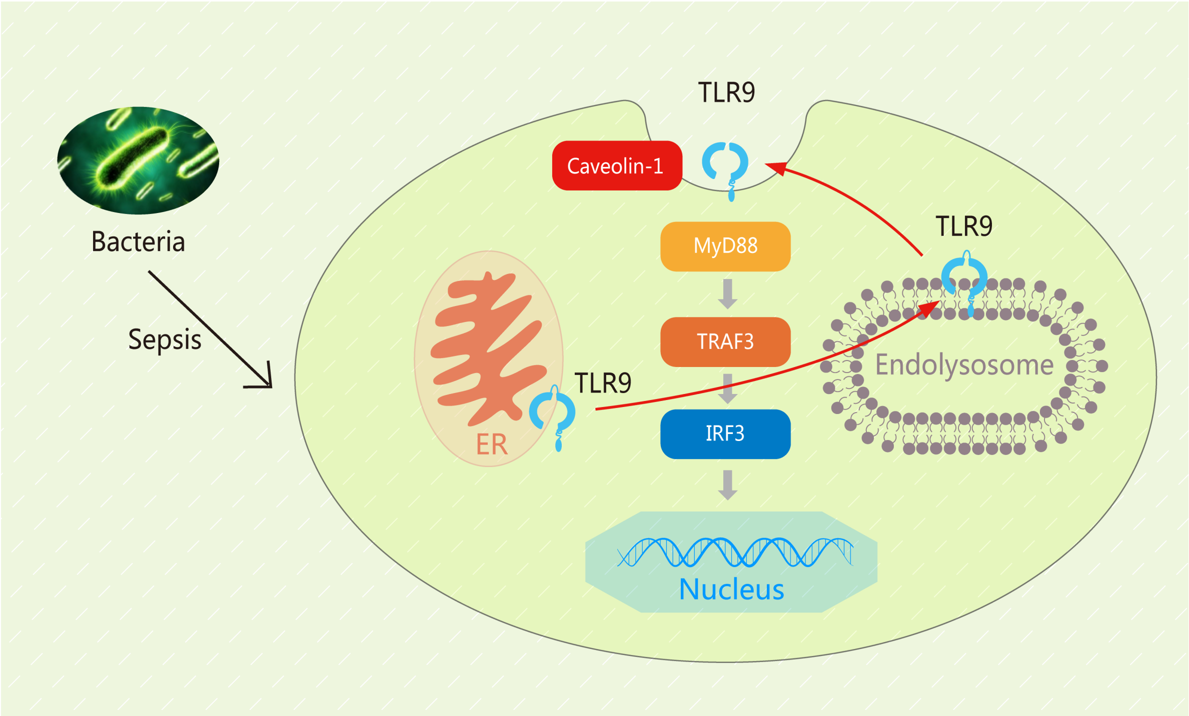

Figure S6. Schematic model of inflammatory preconditioning (InP). Bacteria are sensed by cell membrane TLR9 in neutrophils. TLR9 can be found in the endoplasmic reticulum (ER), on the cell membrane, and in the endolysosome. TLR9 can translocate from the ER to the cell membrane or endolysosome. In neutrophil membranes, membrane TLR9 can bind with Cav-1 and activate downstream signaling pathway members MyD88, TRAF3, and IRF3.

Supplemental Table S1: PCR primer sequences

Gene	Forward	Reverse
TLR9	5'-ATGGACGGGAACTGCTACTAC-3'	5'-CATTGGTGTGGGTGATGCTTT-3'
TLR4	5'-AGCCTTTCAGGGAATTAAGCTC-3'	5'- TCCCAAGATCAACCGATGGAC-3'
MyD88	5'-TCGCAGTTTGTTGGATGCCTG-3'	5'-TGTAAAGGCTTCTCGGACTCC-3'
IRAK4	5'-CGAGAAGAAAAACAGACGGC-3'	5'-CAAAGTGCTTTTCCGGTCTC-3'
TRAF3	5'-ACTGAGCTGGAGAGCGTAGA-3'	5'-GCGCTTGTAGTCACGGATCT-3'
TRAF6	5'-GAATCACTTGGCACGACACTT-3'	5'-GAGTTTCCATTTTGGCAGTCA-3'
P38	5'-CTATGGCTCGGTGTGTGCT-3'	5'-GCTGCACACACTATTCCTTGAG-3'
IRF3	5'-GATGGAGAGGTCCACAAGGA-3'	5'-GAGTGTAGCGTGGGGAGTGT-3'
IRF7	5'-TCGTGATGCTGCGGGATAACT-3'	5'-AGCATGTGTGTGTGCCAGGAA-3'
TNF-α	5'-GACCCTCACACTCAGATCATCT-3	5'-TCCCTTCACAGAGCAATGACTC-3'
NFκB	5'-GCATTCTGACCTTGCCTAT-3'	5'-CCAGTCTCCGAGTGAAGC-3'

Supplemental Table S2: Patient information

	Healthy (Mean+SD)	Recovery (Mean+SD)	Dead (Mean+SD)
Age, years			
$\leq$ 65	15 (45.1+13.2)	13 (51.4+11.0)	12 (51.8+9.0)
>65	15 (71.8+5.6)	15 (74.9+6.9)	13 (74.7+7.4)
Sex			
Male	15	14	17
Female	15	14	8
White blood cell counts	7.1+1.2	17.2+7.7	22.1+27
Blood C-reactive protein levels	2.7+1.4	123.7+81.9	137+135.7
Procalcitonin blood levels	0.2+0.1	19.0+30.2	11.3+10.5

Supplemental Table S3: ROC Curves data

	Membrane TLR9	Cav-1
Sensitivity	0.667	0.481
Specificity	0.788	0.731
Cutoff	50785	73175
95%CI for the AUC	0.616-0.860	0.447-0.720
